# Genomic landscape of HIV-1 proviruses in ART-treated immunological non-responders

**DOI:** 10.3389/fimmu.2026.1804632

**Published:** 2026-08-12

**Authors:** Shengquan Tang, Yue Wang, Yanqiu Lu, Zhen Tong, Jiahui Guo, Yizhi Cui, Vijay Harypursat, Yemiao Chen, Jing Ouyang, Huan Li, Tong Wang, Quanhua Zhou, Yaokai Chen

**Affiliations:** 1 Department of Infectious Diseases, Chongqing Public Health Medical Center, Chongqing, China; 2 Basic Medicine College, Chongqing Medical University, Chongqing, China; 3 Chongqing Key Laboratory of Highly Pathogenic Microbes, Chongqing Research Center for Disease Prevention and Public Health, Chongqing Center for Disease Control and Prevention (Chongqing Academy of Preventive Medicine), Chongqing, China; 4 The First Affiliated Hospital, MOE Key Laboratory of Tumor Molecular Biology, College of Life Science and Technology, Jinan University, Guangzhou, Guangdong, China

**Keywords:** HIV reservoir, immunological non-responders, intact and defective provirus, near full-length sequencing, proviral integrity

## Abstract

**Background:**

Immunological non-responders (INRs) and immunological responders (IRs) exhibit distinct patterns of immune reconstitution despite suppressive antiretroviral therapy (ART); however, the genetic characteristics of the HIV-1 reservoir in these groups remain poorly understood. This study investigates the landscape of HIV-1 proviruses in INRs and IRs, and its potential association with immune reconstitution.

**Methods:**

Peripheral blood mononuclear cells (PBMCs) from 10 ART-treated INRs and 10 IRs were analyzed using near-full-length HIV-1 DNA amplification and PacBio third-generation sequencing to characterize genetically intact and defective proviral genomes. Intact open reading frames (ORFs) were further inferred using the CFEIntact tool.

**Results:**

Compared with IRs, INRs had a higher total proviral genome per 10^6^ PBMCs, although the difference did not reach statistically significance. INRs also displayed a significantly higher level of intact proviral genomes per 10^6^ PBMCs and a trend toward a higher proportion of intact sequences. Defective proviruses remained the predominant component of the reservoir in both groups and were broadly comparable in number and composition. In both cohorts, proviral defects were more frequently observed in the 3′ half of the genome.

**Discussion:**

Our study reveals a distinct proviral landscape in ART-treated INRs, characterized by elevated levels of intact proviral genomes. These observations suggest that the intact component of the reservoir may represent a clinically relevant feature of incomplete immune reconstitution, and provide a rationale for further studies of reservoir-directed interventions in this population.

## Introduction

Current ART effectively suppresses HIV replication and increases CD4^+^ T-lymphocyte counts; however, it cannot eradicate the HIV-1 reservoir. Persistent viral reservoirs can intermittently release low levels of viral antigens and viral products, which may act as chronic stimuli that sustain immune activation and inflammation, even during virologic suppression (1, 2). This ongoing immune activation triggers a cascade of pathological processes: it induces sustained proliferation and exhaustion of CD4^+^ T-lymphocytes, impaired thymic output of naïve T cells, and disruption the intricate balance of immune homeostasis. Over time, these perturbations may lead to incomplete recovery of CD4^+^ T-lymphocyte counts despite undetectable plasma viral loads, a state referred to as immunological non-response. Compared with IRs, INRs are more prone to opportunistic infections and non-AIDS-defining events (NADEs) (3), and have higher mortality rates (4).

HIV proviruses, which may be categorized into intact and defective forms (5), refer to viral DNA integrated into the host genome. Genetically intact proviruses may retain the capacity to produce replication-competent virions (6). The majority of proviruses are defective, and these defective proviruses accumulate rapidly within the first few weeks of infection and eventually account for over 90% of the total proviral pool following ART (5, 7, 8). While early studies suggested that defective proviruses lack biological functions, recent research has demonstrated that they may still produce unspliced HIV-1 RNA and viral proteins. This process trigger both innate and adaptive immune responses, leading to persistent immune activation, inflammation, and multiorgan damage (9). Although INRs and IRs exhibit different immune reconstitution outcomes, little is known about the differences in their HIV-1 reservoirs at the genomic level. In particular, the structural integrity of proviral genomes across distinct HIV-1 subtypes and its potential implications for immune reconstitution and viral rebound remain poorly understood. Our previous investigations have demonstrated that INRs exhibit significantly higher total HIV-1 DNA and HIV-1 cell-associated RNA (CA-HIV RNA) levels compared to IRs (10). However, the relative contributions of intact versus defective proviral reservoirs to this disparity remain to be explored in detail.

In this study, we aimed to compare the genetic integrity of HIV-1 proviruses in PBMCs from INRs and IRs, using near-full-length HIV-1 DNA amplification and third-generation sequencing to characterize the prevalence of intact and defective genomes. By defining the genomic landscape of HIV-1 reservoirs in these two groups, we aim to shed light on the potential mechanisms that underlie immune reconstitution failure and the persistence of viral reservoirs in INRs.

## Materials and methods

### Study population and samples

All individuals living with HIV were recruited from patients seen at Chongqing Public Health Medical Center (CPHMC), China. Written informed consent was obtained from all participants, and the study protocol was approved by the institutional ethics committee of CPHMC. The inclusion criteria for INRs and IRs were as follows: age >18 years; receipt of continuous ART for at least 24 months; and achievement and maintenance of virological suppression, defined as a plasma viral load <50 copies/mL or below the lower limit of detection. Exclusion criteria included the presence of any opportunistic infections, autoimmune diseases, active chronic hepatitis B or C infection, malignancies, or active tuberculosis. Participants with CD4^+^ T-lymphocyte counts <200 cells/μL after at least 36 months of continuous ART were classified as INRs, whereas those with CD4^+^ T-lymphocytes counts >500 cells/μL were classified as IRs. Peripheral blood mononuclear cells (PBMCs) were isolated from whole blood using lymphocyte separation medium (TBD Sciences, China) according to the manufacturer’s instructions, and were cryopreserved in liquid nitrogen until further use.

### Nucleic acid extraction

Total nucleic acids were extracted from 1×10^6^ peripheral blood mononuclear cells (PBMCs) using the DNeasy Blood & Tissue Kit (QIAGEN, Germany) according to the manufacturer’s protocol. Nucleic acids were eluted from the spin column with 100 μL of DEPC-treated water. The concentration and purity of DNA were determined using a NanoDrop 2000 spectrophotometer (Thermo Fisher Scientific, USA). All samples were stored at −80 °C, and repeated freeze–thaw cycles were avoided.

### Amplification of near-full HIV DNA

Near-full-length HIV-1 DNA was amplified using a published method, with minor revision (11). The sequences of the primers for the two rounds of PCR reactions were as follows: first-round forward primer: 5′-AAATCTCTAGCAGTGGCGCCCGAACAG-3′, first-round reverse primer: 5′-TGA-GGGATCTCTAGTTACCAGAGTC-3′, second-round forward primer: 5′-GCGCCC-GAACAGGGACYTGAAARCGAAAG-3′, second-round reverse primer: 5′-GCAC-TCAAGGCAAGCTTTATTGAGGCTTA-3′. These two pairs of primers correspond to base pair (bp) positions 623–649, 9,662–9,686, 638–666, and 9,604–9,632 of the standard reference sequence HXB2, respectively. The parameters for the two rounds of nested PCR amplification were identical and as follows: 94 °C for 2 min, 10 cycles of 94 °C for 10 s, 60 °C for 30 s, and 68 °C for 10 min, 20 cycles of denaturation at 94 °C for 10 s, 55 °C for 30 s, and extension at 68 °C for 10 min, and further extension at 68 °C for 10 min. A 16-bp barcode was attached to the 5’ end of both the forward and reverse primers to distinguish different samples. Positive reactions were determined by visualization of amplicons on 1% agarose gel. All amplicons were sequenced using the PacBio sequencing platform.

### Data analysis

Viral genome intactness was assessed using the HIVSeqinR bioinformatics pipeline (12). Briefly, any sequence with ≥80% of its total contig length mapped to HXB2 was defined as HIV. Genomic classifications were then assigned as follows: “LargeDeletion” refers to any genome with a length of <8000 base pairs, “Internal inversions” denotes any genome containing internal inversions, “Scramble” describes any genome with out-of-order regions, “Hypermut” refers to any genome harboring APOBEC-3G/3F-associated hypermutations, “PrematureStop” indicates any genome with a premature stop codon in the *gag*, *pol*, or *env* gene, and “5DEFECT” is defined as any genome with 15-nucleotide insertions or deletions relative to the reference sequences HXB2 and NL4–3 within genomic coordinates 638–789, while genomes lacking all the preceding defects are classified as “intact” (12, 13).

### Integrity analysis

HIV-1 genomic integrity was assessed using CFEIntact (https://github.com/cfe-lab/CFEIntact) across nine coding regions (*gag*, *pol*, *vif*, *vpr*, *tat*, *rev*, *vpu*, *nef*, and *env*) and two regulatory elements (Ψ and RRE). Briefly, the tool assigns a subtype to each input sequence via BLAST and performs a global pairwise alignment to the corresponding subtype reference. A coding region was considered intact if its open reading frame (ORF) was present and free of deletions, insertions, frameshifts, internal stop codons, or mutated start/stop codons. The Ψ and RRE elements were considered intact in the absence of predicted packaging-signal or RRE defects, respectively.

### Statistical analysis

Continuous variables were summarized as medians with interquartile ranges (IQRs), whereas categorical variables were presented as counts and percentages. Group comparisons for categorical variables were performed using Fisher’s exact test or the Chi-square test, as appropriate. Between-group comparisons for continuous variables were performed using the Wilcoxon rank-sum test. Correlations were assessed using Spearman’s rank correlation coefficient. All statistical analyses and data visualizations were conducted using R software (version 4.1.2, University of Auckland, New Zealand) and GraphPad Prism (version 6.02, GraphPad Software, San Diego, California, USA). A two-sided *P*-value < 0.05 was considered statistically significant.

## Results

### Study participants

**Figure 1:**
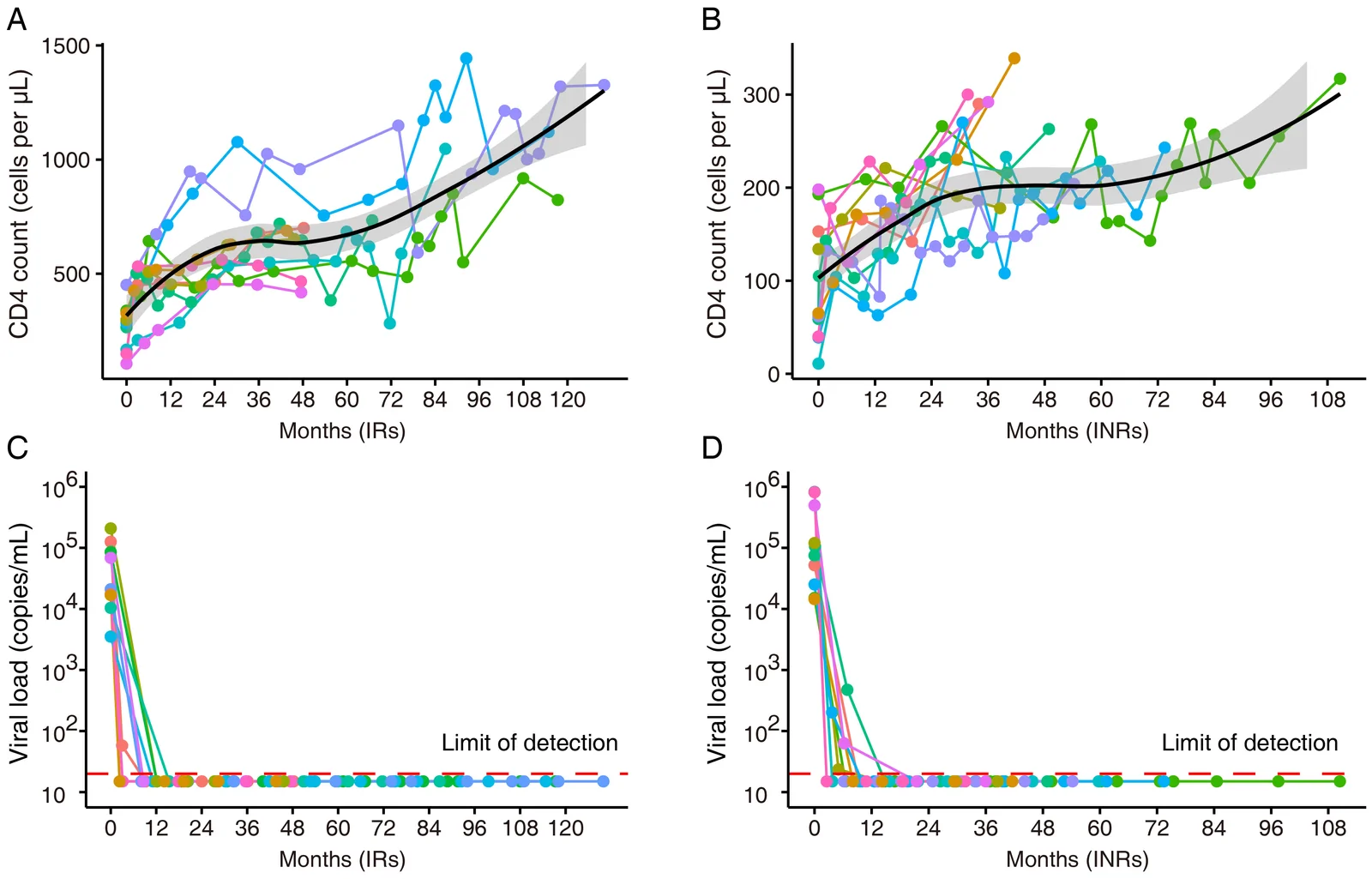
Longitudinal changes in CD4^+^ T-lymphocyte counts and viral loads among HIV-1 infected participants on ART. A total of 10 IRs **(A, C)** and 10 INRs **(B, D)** are shown. Each color denotes an individual participant. Solid circles indicate observed data points. The black line represents the fitted curve, and the gray shading indicates the 95% confidence interval.

A total of 10 IRs and 10 INRs were enrolled in this study according to the criteria outlined in the Methods section (**Table 1**). For INRs, the median duration of ART was 3.8 years (Interquartile range, IQR: 3.1, 4.9, **Figure 1D**). Their median CD4^+^ T-lymphocyte count at the initial measurement was 64 cells/μL (45, 148), which subsequently increased to 173 cells/μL (85, 203); however, none of the INRs exceeded the upper threshold of 350 cells/μL defined for INR enrollment at the time of inclusion (**Figure 1B**; **Table 1**). In contrast, the median ART duration for IRs was 4.8 years (4.0, 9.0) (**Figure 1C**), and their CD4^+^ T-lymphocytes counts rose from 291 cells/μL (192, 334) to 606 cells/μL (567, 714) (**Figure 1A**; **Table 1**). Unsurprisingly, the median CD4^+^ T-lymphocyte counts (both pre- and post-ART) were significantly lower in INRs compared to IRs (**Table 1**). Additionally, INRs exhibited a lower CD4/CD8 ratio than IRs, both pre- and post-ART. However, the CD8^+^ T-cell counts were comparable between the two groups. No significant differences were observed between INRs and IRs in terms of gender, age, baseline ART regimen, or transmission route (**Table 1**).

**Table 1:** Clinical information of the 10 INRs and 10 IRs.

Characteristics of study population	IR (N = 10)	INR (N = 10)	*P*
Age (years)	36 (31, 43)	43 (33, 47)	0.31
Men (n, %)	9 (90.0)	8 (80.0)	1.00
Months on treatment	57.6 (46.6, 111.6)	45.2 (37.1, 66.6)	0.10
Pre-ART HIV-1 RNA (log10 copies/mL)	4.3 (4.1, 5.0)	5.0 (4.4, 5.9)	0.15
Pre-ART CD4^+^ T-cell count (cells/µL)	291 (192, 334)	64 (45, 148)	<0.01
Post-ART HIV-1 RNA (copies/mL)	Below detection limit	Below detection limit	–
Post-ART CD4^+^ T-cell count (cells/µL)	606 (567, 714)	173 (85, 203)	<0.01
Post-ART CD8^+^ T-cell count (cells/µL)	705 (500, 1191)	590 (493, 748)	0.16
Post-CD4^+^/CD8^+^ ratio	0.91 (0.66, 1.12)	0.25 (0.16, 0.35)	<0.01
ART regimens at initial stage			1.00
2 NRTIs + 1 NNRTI	8 (80.0)	7 (70.0)	
2 NRTIs + 1 PI	2 (20.0)	3 (30.0)	
Mode of transmission			1.00
Heterosexual	9 (90.0)	10 (100.0)	
Undetermined	1 (10.0)	0	

### Dominant sequence characterization of HIV-1 DNA in INRs and IRs

In the present study, sequences with a frequency exceeding 50 were defined as valid sequences, whereas the top 20 most frequent amplicons were designated as dominant sequences for each sample (14). Using the predominant subtypes in China as reference sequences (15), the dominant sequences from each sample were aligned with these references. Subsequently, a phylogenetic tree was constructed using FastTree (version 2.1.11) with an approximate maximum-likelihood algorithm characterize the distribution features of the dominant sequences of HIV-1 DNA amplicons in INRs and IRs.

**Figure 2:**
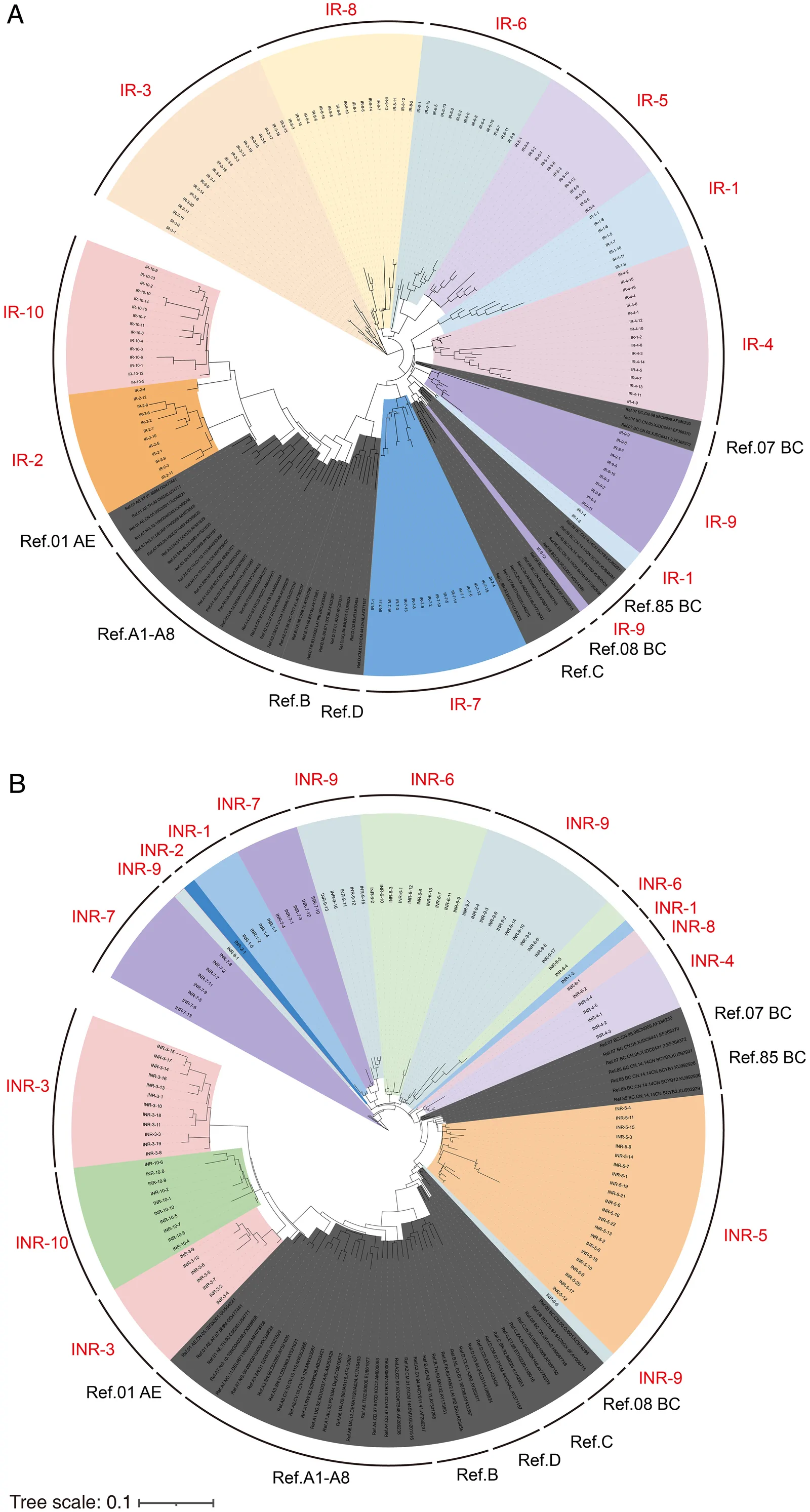
Phylogenetic analysis. Phylogenetic tree of HIV-1 DNA of IRs **(A)** and INRs **(B)**. Participants are distinguished by unique colors. The reference sequence is shown in dark gray.

A total of 144 HIV-1 DNA dominant sequences were identified from 10 IRs, belonging to the CRF01_AE, CRF07_BC, CRF08_BC, CRF85_BC, and D subtypes. Specifically, six IRs were classified as CRF07_BC, two as CRF85_BC, and two as CRF01_AE, with one case each of CRF08_BC and D (**Figure 2A**). Moreover, the coexistence of multiple HIV variants was observed in two patients: IR-1 harbored sequences belonging to CRF07_BC and CRF85_BC, while IR-9 contained sequences belonging to CRF08_BC and CRF85_BC (**Figure 2A**). Meanwhile, 107 HIV-1 DNA dominant sequences were identified from 10 INRs. Consistent with the subtype distribution in IRs, these sequences belonged to the CRF01_AE, CRF07_BC, CRF08_BC, and CRF85_BC subtypes. Specifically, sequences from seven INRs were classified as CRF07_BC, two as CRF01_AE, and one as CRF85_BC (**Figure 2B**). Among INR participants, coexistence of sequences was observed only in INR-9, who harbored sequences belonging to CRF07_BC and CRF08_BC (**Figure 2B**).

### Comparison of HIV-1 genome integrity between IRs and INRs

**Figure 3:**
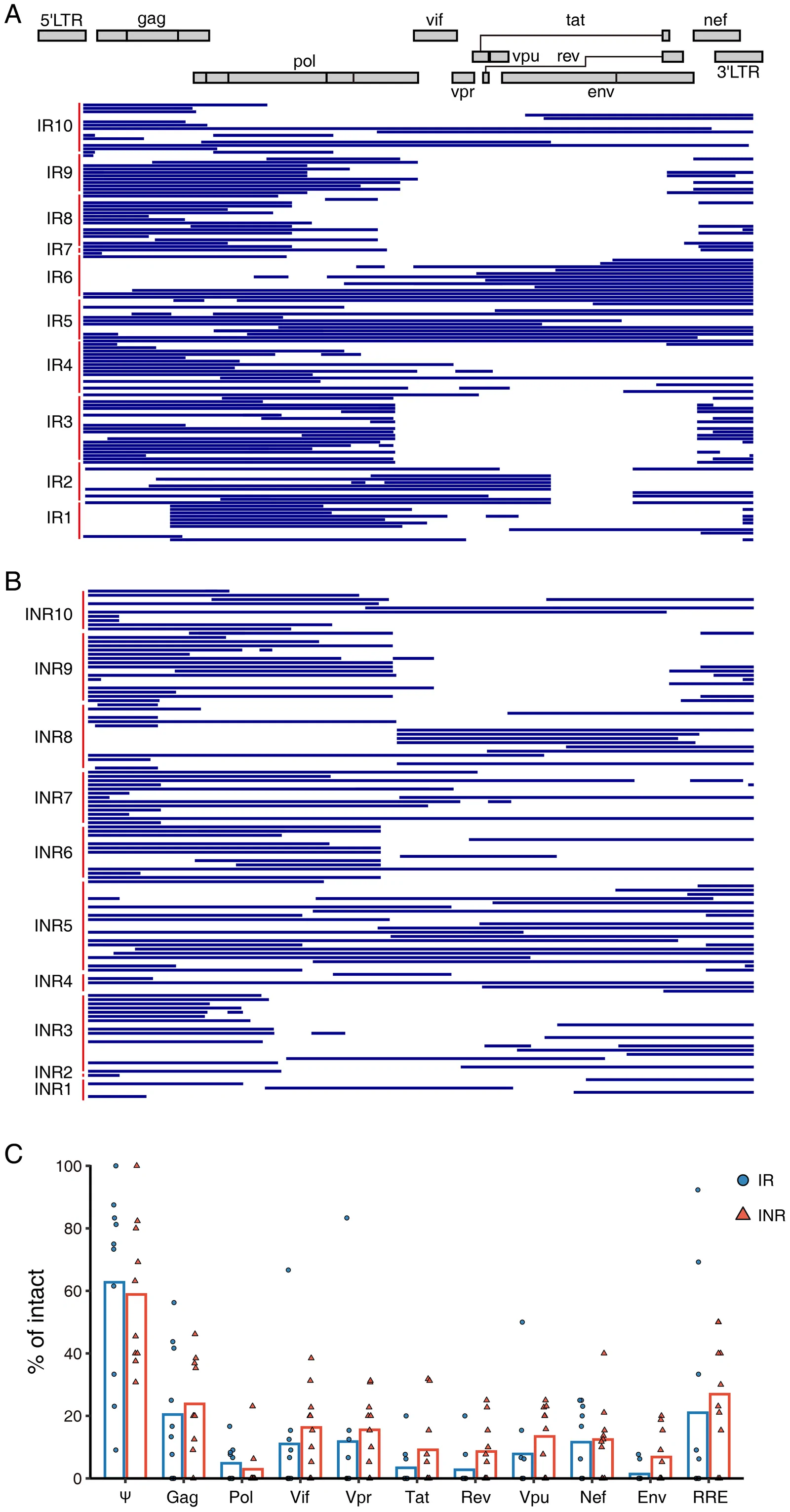
Integrity analysis of HIV-1 proviral genomes from IRs and INRs. Virograms showing dominant sequence characterization of HIV DNA PCR products amplified from IRs **(A)** and INRs **(B)**. The genome structure and sequence coordinates of HIV-1 HXB2 are shown at the top as reference. **(C)** The proportions of proviruses with intact Ψ, gag, pol, vif, vpr, tat, rev, vpu, nef, env, and RRE (Rev response element) were compared between IRs and INRs. Each dot represents one participant. Differences in the percentage of intact coding regions or regulatory elements between IRs and INRs were assessed using the nonparametric two-sided Mann–Whitney U test.

We next compared HIV-1 genome integrity between IRs and INRs. Proviral defects have been reported to preferentially affect the 3′ half of the genome, particularly *env, tat, rev*, and *nef* (5, 16, 17). Consistent with this positional bias, both cohorts exhibited lower intact proportions in several 3′ coding regions (most notably *env*) than in the 5′ region gag (**Figures 3A–C**). In a region-by-region analysis of all nine coding genes and two regulatory elements (Ψ and RRE), Ψ was retained at relatively high proportions in both groups (**Figure 3C**). INRs tended to harbor higher intact proportions of *gag, vif, vpr, tat, rev, vpu, nef, env*, and RRE, whereas pol and Ψ showed comparable levels between the groups. However, none of these region-specific differences reached statistical significance (**Figure 3C**).

### Intact proviral genomes were significantly more abundant in INRs than IRs

**Figure 4:**
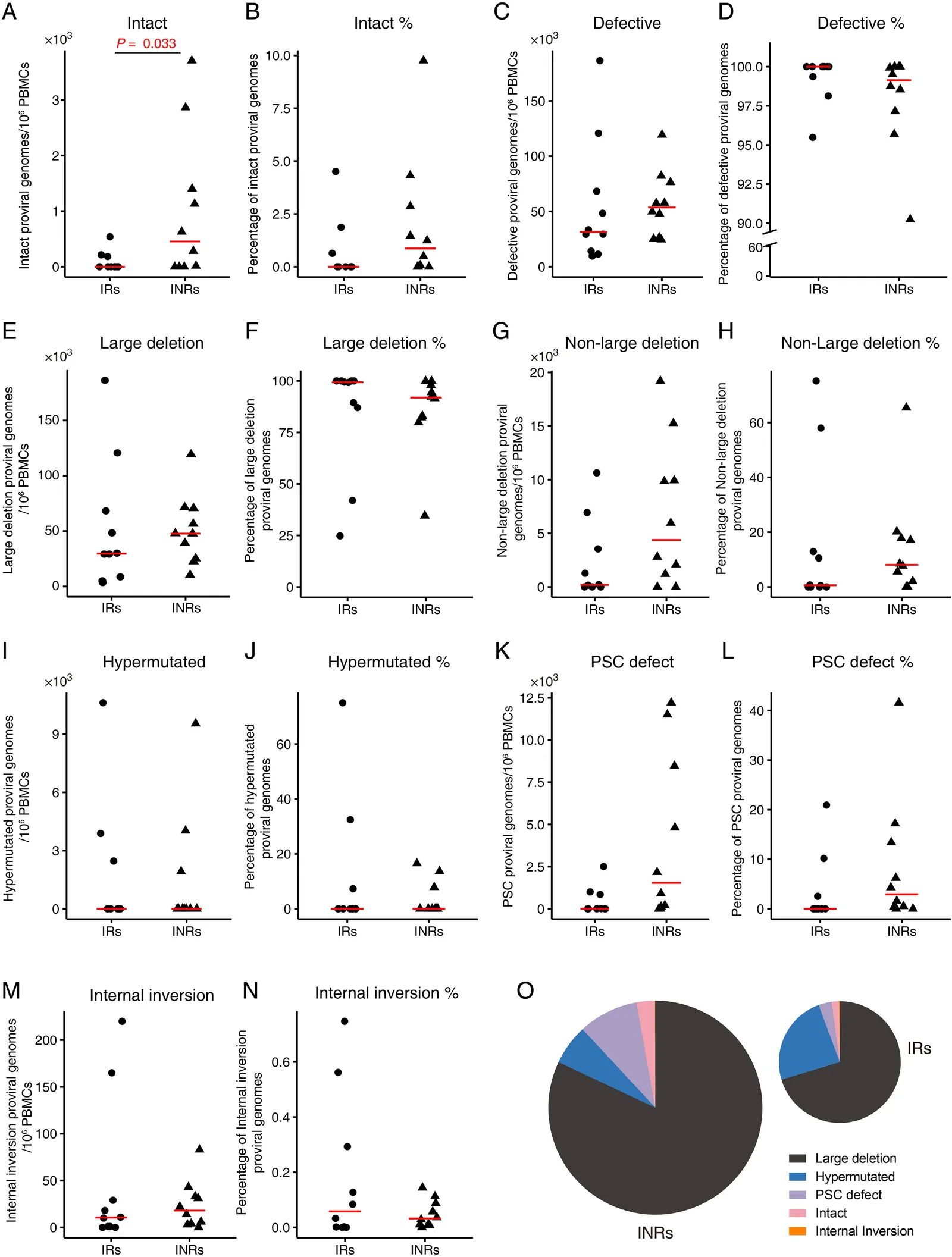
Comparison of reservoir measures between IRs and INRs. **(A–N)** Comparison of copy numbers and proportions of different proviral genome types within the total reservoir between groups. **(O)** Pie charts illustrate the median proportional distribution of proviral species across all participants, normalized to 100%. Statistical significance was assessed using a Mann–Whitney U test.

To date, the precise composition of the intact proviral reservoir in INRs has not been well defined. We hypothesized that INRs may harbor a larger reservoir of genetically intact provirus post ART. Supporting our hypothesis, intact proviruses were detected in a greater proportion of INRs than in IRs (7/10 vs. 3/10; **Figures 4A, B**). Moreover, the median level of intact proviral genomes was significantly higher in INRs than in IRs (455 copies/10^6^ PBMCs [IQR: 8.5, 1922.8] vs. 0 copies/10^6^ PBMCs [IQR: 0, 92.5], *P* < 0.05, **Figure 4A**). The proportion of intact proviruses relative to total proviruses was also higher in INRs than in IRs, although this difference did not reach statistical significance (*P* = 0.14, **Figure 4B**). In addition, the median total HIV DNA level was higher in the INR group than in the IR group, at 54,079 versus 31,535 copies/10^6^ PBMCs, however, this difference was not statistically significant (*P* = 0.43). Collectively, these findings indicate that INRs in this cohort harbor higher levels of genetically intact proviral genomes despite sustained viral suppression.

Consistent with previous reports (5, 7), defective proviruses accounted for the vast majority of the total proviral reservoir, representing median proportions of 99.3% in IRs and 91.9% in INRs (**Figure 4O**). However, neither the counts nor the percentages of defective proviral genomes differed significantly between the two groups (**Figures 4C, D**). Among the various categories of defective proviruses, large-deletion HIV-1 DNA (defined as viral sequences shorter than 8,000 bp) was the most abundant subtype in both cohorts. Further comparison showed no significant differences in either the counts or proportions of large-deletion proviruses between INRs and IRs (**Figures 4E, F**). We next examined whether non–large-deletion proviral genomes (≥8,000 bp), which may retain more intact open reading frames (ORFs), were more frequent in INRs. However, no significant differences were observed between the two groups in either the counts or the proportions of these genomes (**Figures 4G, H**). In addition, no significant differences were observed between IRs and INRs in either the absolute counts or the proportions of hypermutated, premature stop codon (PSC)–defective, and internal inversions proviral genomes (**Figures 4I–N**).

## Discussion

In this study, the majority of the dominant sequences in both the IRs and INRs cohorts belong to the CRF07_BC subtype, consistent with our previous findings. Specifically, this previous research, conducted from 2018 to 2021 and enrolling 1,110 HIV-infected patients from Chongqing, revealed that the CRF07_BC subtype accounted for 55.68% of the viral strains in the local HIV-infected population (15). Distinct HIV-1 subtypes exert significant effects on the recovery of CD4^+^ T-lymphocyte counts in individuals receiving ART. In a retrospective cohort study, Li et al. followed 403 HIV-1-infected patients undergoing ART for 15 years, and employed Cox proportional hazards models and propensity score matching to evaluate factors influencing immune reconstitution. Their observations revealed that patients infected with the CRF01_AE strain exhibited a significantly shorter median time from ART initiation to achieving immune reconstitution compared to those infected with other subtypes or subclusters (3). In a separate investigation, Tarosso et al. enrolled 68 patients infected with HIV-1 subtype B and 10 patients with subtype BF recombinant. Even when plasma viral loads were comparable between the two groups, patients infected with the BF subtype displayed a more rapid decline in CD4^+^ T-lymphocyte counts, with a monthly reduction rate of 6.3 cells/μL, whereas the decline rate in subtype B-infected patients was 3.6 cells/μL (18). These observations collectively highlight the subtype-specific variations in immune dynamics during HIV-1 infection and ART management. However, in the present study, no significant differences were observed in the distribution of either CRF07_BC or other subtypes between IRs and INRs.

We observed a significantly higher absolute number of intact proviral genomes in PBMCs from individuals with INRs than in those from IRs. We also observed a higher percentage of intact proviral genomes in INRs, although this comparison did not reach statistical significance. These findings are consistent with a study by Muñoz-Muela et al., who reported higher intact HIV-1 DNA levels in INRs than in IRs (46 vs. 9 copies/10^6^ monocytes) (19). However, Scrimieri et al. observed no significant difference in intact HIV-1 DNA levels between IRs and INRs in PBMCs using near-full-length HIV-1 sequencing (intact/total sequences: 22/423 vs. 11/253, respectively) (20). Direct comparison of our findings with those reported by Scrimieri et al. should be interpreted cautiously because of differences in several aspects, including: (i) sequencing approaches, as Scrimieri et al. used near-full-length HIV-1 sequencing, whereas our study applied third-generation long-read sequencing; (ii) clinical definitions of immune recovery status, as Scrimieri et al. classified IRs and INRs using CD4+ T-cell thresholds of ≥250 and <250 cells/μL, respectively, whereas our study adopted more stringent criteria (≥500 and <200 cells/μL, respectively); and (iii) criteria used for proviral intactness assessment, as different annotation strategies may influence the classification and quantification of intact proviral genomes. Collectively, these observations highlight that the relationship between intact HIV reservoirs and incomplete immune recovery remains an important but incompletely understood question. By comprehensively characterizing proviral integrity in IR and INR populations, our study provides additional insight into reservoir characteristics associated with incomplete immune recovery.

In our cohort, INRs harbored a higher level of bioinformatically inferred intact proviruses, suggesting a reservoir with greater transcriptional and antigenic potential. This is relevant because residual HIV transcription and viral protein expression can persist despite suppressive ART, sustaining ongoing antigenic stimulation (9, 21, 22). Such residual viremia has been associated with persistent T-cell activation in INRs and with distinct immune activation profiles during suppressive ART (23, 24), and chronic immune activation is itself linked to impaired CD4^+^ T-cell recovery (25, 26). Mechanistically, HLA-DR^+^ memory CD4^+^ T cells are enriched for intact HIV genomes, while PD-1/TIGIT/LAG-3-expressing CD4^+^ subsets harbor persistent, inducible virus (27, 28). Moreover, both total and intact HIV DNA have been positively correlated with T-cell activation and exhaustion, whereas HIV-specific dual-cytokine CD4^+^ responses are inversely associated with intact proviral proportions during long-term ART (PMID: 38014340). Collectively, these findings support a biologically plausible link between a transcriptionally active reservoir, persistent immune activation, and poorer CD4^+^ T-cell recovery, with HIV-specific T-cell dysfunction/exhaustion as a related feature. However, we acknowledge that our cross-sectional study design cannot determine whether higher intact proviral burden is mechanistically upstream of the INRs phenotype or whether both are downstream consequences of more advanced pre-ART immune injury.

Intact HIV-1 proviral DNA, in contrast to its defective counterparts, exhibits a modest decrease in frequency following the initiation of ART (29–32). Emerging evidence further indicates that intact proviral decay under suppressive ART is heterogeneous across individual (33), and that lower pre-ART or nadir CD4+ T-cell counts are associated with a smaller decline in intact proviral DNA over time (31, 32). This association is biologically plausible because CD4+ T cells coordinate antiviral immunity and support the recognition and elimination of virus-producing cells after reactivation (34, 35). Because a low pre-ART CD4+ T-cell count is a defining characteristic of INRs (36), profound CD4+ T-cell depletion before ART may weaken immune clearance of reactivated infected cells, thereby contributing to slower decay of intact proviruses. The observed differences in intact proviral genome levels between IRs and INRs may therefore be attributable, at least in part, to differences in intact proviral decay rates, which may be modulated by a complex interplay of host immune factors, viral characteristics, and the cellular microenvironment of the viral reservoir. Longitudinal monitoring of intact proviruses before and after ART initiation in INR populations will be needed to further clarify the relationship between intact proviral persistence and CD4+ T-cell recovery.

Consistent with previous reports (5, 7, 22, 37, 38), we observed that the majority of HIV proviral genomes amplified from participant-derived DNA were defective in both INRs and IRs. This finding corroborates those reported by Scrimieri et al., who conducted comprehensive analyses of 24 IRs and 22 INRs individuals, revealing that defective HIV-1 DNA accounted for 94.8% and 95.7% of the viral reservoir, respectively (20). This pattern was observed in participants treated during both the early and chronic stages of HIV infection. Defective proviruses, although incapable of generating infectious virions, can still produce various HIV-1 transcripts and proteins. As a result, they may contribute to the deleterious chronic inflammation and immune activation seen in individuals with HIV-1, even with effective ART (39, 40). These proteins may be secreted in exosomes (41) or in soluble forms, potentially mediating apoptosis in both infected and uninfected cells (42).

At the gene level, both IRs and INRs showed a shared positional bias in proviral genome integrity, with several regions in the 3′ half being more frequently defective than those in the 5′ half, particularly env, consistent with previous reports (5, 16, 17). Given that Tat and Rev are essential for HIV transcription and RNA export (43), defects in these regions may substantially limit viral gene expression. Although INRs tended to show higher intact proportions across several coding regions and RRE, none of the region-specific differences between INRs and IRs reached statistical significance.

It is important to acknowledge the limitations of the present study. Our sample size was relatively small, and the single-time-point design precludes definitive conclusions about whether the observed differences in intact proviral burden represent stable characteristics of INRs versus IRs over time. This represents a primary limitation of the present study. Therefore, larger longitudinal cohorts will be needed to validate these findings and to evaluate the temporal dynamics of intact and defective proviruses in INRs and IRs. Additionally, our study did not assess the integration status or replication competence of HIV DNA. Research on CD4^+^ T-lymphocytes from individuals who naturally control HIV-1 infection has demonstrated that intact proviruses are predominantly located in heterochromatic regions of the host genome, whereas defective proviruses are detected in euchromatic regions (1, 44, 45). Analyzing the integration sites may potentially help elucidate the reasons behind the higher prevalence of intact proviruses in INRs.

Collectively, using near-full-length HIV-1 DNA amplification and PacBio sequencing, we found that INRs harbor a significantly higher absolute number of intact proviral genomes than IRs, with a trend toward a higher proportion of intact sequences, whereas defective proviruses remained the dominant component of the reservoir in both groups and were broadly comparable in number and composition. At the gene level, both groups showed a shared positional bias in proviral genome integrity, with defects more frequently affecting regions in the 3′ half of the genome. The higher intact proviral levels in INRs may reflect slower decay rates, potentially linked to their lower pre-ART CD4^+^ T-lymphocyte counts and impaired immune clearance of reactivated infected cells. These observations suggest that INRs possess a reservoir not only larger in size, but also distinct in genetic composition, with potential implications for persistence and rebound risk. Larger studies incorporating integration site mapping and functional assays are warranted to confirm these results and clarify their implications for HIV cure strategies.

## Data Availability

The datasets presented in this study can be found in online repositories. The names of the repository/repositories and accession number(s) can be found below: https://ngdc.cncb.ac.cn/search/specific?db=bioproject&q=PRJCA044554, PRJCA044554.
